# Milder presentation of autosomal dominant fatty acyl CoA reductase 1‐related syndrome: Report of the first Middle Eastern patient and review of the literature

**DOI:** 10.1002/ccr3.6307

**Published:** 2022-10-13

**Authors:** Mohammed Almuqbil, Ahlam AbuMelha, Daniah Albokhari

**Affiliations:** ^1^ College of Medicine King Saud bin Abdulaziz University for Health Sciences (KSAU‐HS) Riyadh Saudi Arabia; ^2^ Division of Pediatric Neurology, King Abdullah Specialist Children’s Hospital (KASCH) National Guard Health Affairs (NGHA) Riyadh Saudi Arabia; ^3^ King Abdullah International Medical Research Center (KAIMRC) Ministry of National Guard Riyadh Saudi Arabia; ^4^ Department of Pediatrics Taibah University College of Medicine Medina Saudi Arabia

**Keywords:** CSPSD, Cataracts, FAR1‐related syndrome, Spastic paraparesis, speech delay, seizures

## Abstract

The FAR1‐related phenotypes caused by the *FAR1* gene encodes the peroxisomal protein fatty acyl‐CoA reductase 1 (FAR1), which is required to reduce fatty acids to fatty alcohols used to form ether‐linked alkyl bonds. Biallelic loss‐of‐function variants have been associated with severe psychomotor developmental delay, seizures, cataracts, growth retardation with microcephaly, and spasticity. However, heterozygous variants in *FAR1* have been recently linked to a rare genetic disorder called cataracts, spastic paraparesis, and speech delay (CSPSD). Here, we present the first Middle Eastern patient with a de novo pathogenic heterozygous variant in *FAR1* identified by exome sequencing (ES) analysis and a detailed overview of the reported clinical phenotypes and genotypes. Our patient represents the milder end of the clinical spectrum, with medication‐free seizures by the first year of life, proper speech and fine motor development, as well as an absence of other previously reported features such as learning difficulties, axial hypotonia, and joint contracture. In addition, she had developmental dysplasia of the hip (DDH) that failed medical management, as well as faltering growth. Our patient adds to the small number of patients recognized to date and expands the clinical spectrum to provide better clinical delineation, improve diagnosis, and develop precision medicine approaches for this disorder.

## Abstract 2

We present the first Middle Eastern patient with a de novo pathogenic variant in *FAR1*, representing the milder end of the clinical spectrum. We aim to highlight the presentation of this rare condition to bring awareness to its possibility in the differential diagnosis of patients with infantile seizure and spasticity to ensure early diagnosis and intervention.

## 1 INTRODUCTION

Recently, two disorders with overlapping features and opposite biochemical phenotypes were identified to be related to the *FAR1* gene.^1^, ^2^ The causative gene encodes fatty acyl‐CoA reductase 1 (FAR1), the enzyme responsible for reducing fatty acids to fatty alcohols, which is essential for wax monoester and ether lipid synthesis.^3^ The stability of FAR1 is dependent on cellular plasmalogen levels.^4^, ^5^ Biallelic loss‐of‐function variants causing *FAR1* deficiency (also known as RCDP type 4, MIM 616154)^6^ are characterized by intellectual disability, cataracts, growth retardation, and seizure disorders without rhizomelia or skeletal changes.^1^ Heterozygous variants in *FAR1* cause the loss of feedback regulation leading to plasmalogen accumulation and increase FAR1 protein levels, causing clinical symptoms similar to those associated with FAR1 deficiency, including spasticity, early‐onset seizures, and cataracts (MIM 619338 ).^2^, ^6^ Here, we report an additional case with a de novo pathogenic variant in *FAR1,* expanding the clinical spectrum of cataracts, spastic paraparesis, and speech delay (CSPSD). In particular, our patient seems to represent the milder end of the clinical spectrum, with resolved epilepsy since the age of one‐year, appropriate speech and fine motor development. She has no features of learning difficulties, hypotonia, or joint contracture.

## 2 CASE REPORT

This is a report of a five‐year‐old Middle Eastern female of healthy and consanguineous (first cousin) parents with no family history and an unremarkable perinatal history. At the age of 4 months, her parents noted generalized tonic seizures with up‐rolling eyes and cyanosis lasting 4 minutes. Similar episodes reoccurred two to three times a month. However, routine electroencephalogram (EEG) was normal. Given the frequency of these episodes, she was started on levetiracetam. Once this anti‐seizure medication (ASM) was started at the age of 6 months, her seizures ceased. She remained seizure‐free and no longer required ASMs by the age of 1 year. She underwent hip imaging in the newborn period showing shallowing of the left acetabulum, and repeated images at 1 year of age clearly showed bilateral developmental dysplasia of the hip (DDH). Her early gross motor development was normal up to one year besides her tip‐toeing. At approximately 15 months, the patient's mother noted gross motor regression of unclear etiology. She became unable to stand or walk alone. She eventually regained her ability to stand alone but not walk independently. At two years, bilateral cataracts were identified and removed surgically with intraocular lens implementation. At that time, she was also referred to orthopedics and neurology clinics. At the age of 3, she was evaluated by both specialties and diagnosed with spastic diplegia, and repeated hip X‐rays showed bilateral subluxed hips with acetabular dysplasia. Physical therapy and gait training were immediately initiated, and a brace was applied. However, she exhibited no improvement, and bilateral pelvic osteotomy and hamstring and adductor release at four years were needed. She consistently exhibited appropriate language development; she babbled at six months, said her first word at 13 months, and spoke in two‐word sentences at two. Currently, she can stand alone and walk using a walker. Her speech, cognitive and fine motor development are continuing to be appropriate for her age, and she is performing well in kindergarten. Faltering growth was noted around the age of five, when she dropped from the 42^nd^ percentile for weight to the 2^nd^ percentile over a 9‐month period, despite adequate caloric intake and no evidence of loss or malabsorption. Her height continues to range between the 11^th^ and 18^th^ percentiles, and her head circumference is above the 85^th^ percentile.

Neurological examination showed grade 2 spastic Achilles tendon based on the Ashworth scale, mild weakness (4/5) in her upper and lower limbs, and clonus in both ankles. She had a scissoring gait with toe walking.

Brain MRI, magnetic resonance spectroscopy (MRS), and spine MRI were normal. The basic metabolic work‐up was unremarkable, and the mitochondrial genome was negative. Exome sequencing (ES) analysis showed the heterozygous de novo pathogenic variant c.1438C>T (p. Arg480Cys) in the *FAR1* gene. The de novo status was confirmed based on trio diagnostic settings.

## 3 DISCUSSION

Here, we report the first Middle Eastern patient with a confirmed autosomal dominant form of FAR1‐related disease. Ferdinandusse et al. identified 3 different heterozygous de novo variants affecting the same arginine at amino acid position 480 of *FAR1*, underlying the newly described autosomal dominant CSPSD in 12 patients.^2^ Our patient’s de novo variant was found again at the 480 site, confirming a markedly increased degree of mutability of this site. The heterozygous variant c.1438C>T (p. Arg480Cys) in our patient has been reported in seven patients; heterozygous variants c.1439G>A (p. Arg480His) has been reported in four patients; and the variant c.1439G>T (p. Arg480Leu) has been reported in one patient.

Including our patient, the total number of CSPSD disorder patients in the literature is 13. Pyramidal tract dysfunction was uniformly involved (100%), including spastic features (13/13), axial hypotonia (7/13), and clonus (6/7). All 13 patients exhibited bilateral cataracts; 5 patients were diagnosed since birth, and 8 patients were detected later in childhood, demonstrating the importance of baseline ophthalmological evaluation at the time of diagnosis and continuous follow‐up thereafter. Early‐onset epilepsy occurred in 9/13 patients and appeared well controlled with ASM. Of the 9 patients, 5 achieved ASM‐free seizures. Gross motor skills were the most affected in all patients, followed by speech delay in 11/13 patients, and dysarthria in 5 patients. Eventually, walking with assistance was achieved in 12/13 patients, and 6 patients achieved fluency in speech. Cognitive impairment was identified in 3/13 patients, although below‐average IQ and memory impairment were identified in 1 patient each. Faltering growth was noted only in our patient, although feeding difficulties, dysphagia, and weight and height parameters below the 3^rd^ percentile on one occasion were reported in 1 patient each. Furthermore, constipation was diagnosed in 5/13 patients. Given the small number of cases, it is difficult to determine whether gastrointestinal and growth problems are a possible feature of this condition. In addition, DDH and worsening spasticity failed medical management due to delayed diagnosis and access to orthopedic care in our case, highlighting the importance of early diagnosis and referral to initiate early intervention. Physical therapy to control spasticity and medical management to treat preexisting conditions may minimize long‐term patient disability and the need for surgical intervention. Macrocephaly was identified in 3 patients, including ours, in contrast to the autosomal recessive inheritance form in which microcephaly is a key feature. Neuroimaging was obtained for all 13 patients, with only mild findings of ventricular prominence, abnormal temporal lobe morphology and benign enlargement of subarachnoid spaces in 1 patient each. Dysmorphic facial features were only seen in 1 patient, including a flat facial profile, full cheeks, deep‐set eyes and absence of incisors. A summary of the clinical manifestations of the 13 patients is presented in Table 1.

**TABLE 1:** Summary of the clinical manifestations of the 13 patients with CSPSD

Author	Present case	S. Ferdinandusse et al^a^											
Patient	P 1	P 2	P3	P4	P5	P6	P 7	P 8	P 9	P 10	P 11	P12	P13
Protein variation	C.1438C>T (p.Arg480Cys)	C.1438C>T(p.Arg480Cys)							c.1439G>A (p.Arg480His)				c.1439G>T (p.Arg480Leu)
Gender	F	F	F	M	F	M	M	F	M	F	M	M	F
Ethnicity	Middle eastern	French	ND	ND	ND	ND	Caucasian	ND	Belgian	ND	ND	ND	ND
Age at the study (years)	5	9	4.5	2.3	5	5	3.5	10	19	6	6.5	4	6.5
Age of onset (months)	4	3	ND; Crawl at 14	3‐4	3	2	10	3	3	10	6	8	4
Developmental Delay	+ G. motor at 15mo	+ G. motor, speech	+ GDD	+GDD dysarthria	+ G. motor, speech dysarthria	+G. motor, speech	+G. motor, speech	+, G. motor dysarthria	+ GDD	+G. motor, speech	+GDD	+ G. motor, speech	+G. motor, speech
Best Development	Walker, N speech and fine motor	Unable to walk. Fluent speech	Walker, 2‐3 words	Walker, a lot of single words	Walk with assistance 2 word‐sentences	Pedal an adaptive bike N speech	Post. walker, WC for long distance 75 words	crutches. 3 to 4 grade levels < chrono. age.	Brace, Completed college	Walker, fluent speech	Walker, simple sentences	Walker, fluent speech	Brace, crutches, fluent speech
Intellectual Disability	‐	‐	+	‐	‐	Below average IQ	‐	+	Learning dif. Memory impairment	‐	+	‐	‐
Seizures/Epilepsy	+ (4mo)	+	‐	‐	+	+	+	+	+	‐	+	‐	+
Seizure Semiology	GTC	GTC	NA	‐	FM, 2ry GTC	TC, FM, AS Still has FS	Complex FS	FM	GTC, AS	NA	GTC	NA	GTC
Epilepsy treatment	LEV; DC at 1Y	VPA	NA	NA	LEV, OXCZ; DC at 2Y	LEV,PHB,TOP; DC at 20mo	ND	PHB: DC later	ND	NA	OXCZ	NA	PHB: DC at 14mo
Brain MRI	N	N	ND	Abnormal temp. lobe, ventricular prominence	N	N	N	N	N	N	N	Benign enlargement of subarachnoid spaces	N
Spasticity (LL)	+	+	+	+	+	+	+	+	+	+	+	+	+
Clonus	+	ND	‐	+	+	ND	ND	ND	ND	ND	+	+	+
Hypotonia	‐	+Axial	+Axial	+Central	+Axial	+Axial	‐	+Axial	‐	+Axial	‐	+Axial	‐
Hip dysplasia	+ at 3Y	ND	ND	ND	ND	+ subluxation, med. femoral H. flatness at 3.5Y, dystonia	ND	ND	ND	ND	ND	‐	ND
EEG	N	N	NA	NA	ND	Abnormal	N	ND	Abnormal	NA	ND	ND	ND
Cataract	+ 2Y	+ 18mo	+ 7Y9mo	+ 7mo	+ congenital	+11mo	+ 2Y9mo	+congenital	+ congenital	+10mo	+6mo	+8mo	+2.5Y
GI/Feeding Problems	‐	+Constipation	+Constipation	‐	ND	+ Constipation	‐	+ Dysphagia constipation	ND	ND	ND	ND	+ Feeding dif., constipation
Faltering growth	+, Relative macrocephaly	‐	ND	±(one occasion <3rd %ile)	ND	ND	ND	ND	ND	ND	‐ Obese; Macrocephaly	ND; Macrocephaly	ND
Other	G. motor regression scissoring	Scoliosis	Joint hypermobility pronated feet	Recurrent OM	G. motor regression	Hip, knee, equinus contractures Subluxing patellae	‐	ADHD, anxiety disorder	Hyperlordosis, ptosis	Dysmorphic facial features	Extra lateral incisor,	Ligamentous laxity, flat feet, scissoring. equinus contractures.	‐

^a^ Patients were arranged based on their molecular findings.

Abbreviations: ADHD, attention deficit hyperactivity disorder; AS, absent seizures, Chrono., chronological; DC, discontinued; Dif., difficulties; F, female; FM, focal motor; FS, febrile seizure; G., gross; GDD, global developmental delay; GTC, generalized tonic‐clonic; H., head; IQ, intelligence quotient; LD, long distance; LEV, levetiracetam; TC, tonic‐clonic; Temp., temporal; TOP, topiramate; LL, lower limb; M, male; Med, medial; Mo, months; N, normal; NA, not applicable; ND, not described; OM, otitis media; OXCZ, oxcarbazepine; PHB, phenobarbital; Post., posterior; WC, wheelchair; VPA, valproate; Y, year

In summary, it is important to report and discuss new cases of rare, newly described syndromes to delineate the phenotype of the condition. Mutations in *FAR1* should be considered in patients with infantile seizure and spasticity even in the absence of a family history to ensure early diagnosis and intervention.

## AUTHOR CONTRIBUTIONS

AA collected the clinical data, and DA drafted the initial manuscript and revised the manuscript. MA provided clinical evaluations and critically reviewed and revised the manuscript.

## CONFLICTS OF INTEREST

The authors have no conflicts of interest to disclose.

## CONSENT

This was not required for this research study.

## COMPLIANCE WITH ETHICS GUIDELINES

This study was performed in accordance with the ethical principles for medical research outlined in the Declaration of Helsinki.

## ANIMAL RIGHTS

This article does not contain any studies with animal subjects.

## Data Availability

The data that support the findings will be available in Centogene company at https://login.centoportal.com/oauth/authorize?response_type=code&client_id=211bd8eb37808d824d9d0d308e53ed83&redirect_uri=https:%2F%2Fwww.centoportal.com%2Fsso&target_uri=%2F following an embargo from the date of publication to allow for commercialization of research findings.
